# Joint similarity nonnegative matrix factorization model for identification of recurrence-related association patterns in tumor

**DOI:** 10.1093/bib/bbaf577

**Published:** 2025-11-03

**Authors:** Jin Deng, Junjie Lan, Ruolan Du, Tao Xu, Kaihan Huang, Lechun Liu, Lin Chen, Yongwei Zhang

**Affiliations:** College of Mathematics and Informatics, South China Agricultural University, No. 483 Wushan Street, Tianhe District, Guangzhou 510642, China; College of Mathematics and Informatics, South China Agricultural University, No. 483 Wushan Street, Tianhe District, Guangzhou 510642, China; College of Mathematics and Informatics, South China Agricultural University, No. 483 Wushan Street, Tianhe District, Guangzhou 510642, China; College of Mathematics and Informatics, South China Agricultural University, No. 483 Wushan Street, Tianhe District, Guangzhou 510642, China; College of Mathematics and Informatics, South China Agricultural University, No. 483 Wushan Street, Tianhe District, Guangzhou 510642, China; College of Mathematics and Informatics, South China Agricultural University, No. 483 Wushan Street, Tianhe District, Guangzhou 510642, China; Department of General Practice, Sun Yat-Sen Memorial Hospital, No. 107 Yanjiang West Road, Haizhu District, Guangzhou 510120, China; College of Mathematics and Informatics, South China Agricultural University, No. 483 Wushan Street, Tianhe District, Guangzhou 510642, China

**Keywords:** tumor recurrence, multimodal association, non-negative matrix factorization, biomarkers

## Abstract

The high recurrence rate of tumor limits the growth of precision medicine, whereas the exploration of correlations in multimodal data enables mining of features linked to tumor recurrence, ultimately identifying prospective biomarkers. Nevertheless, existing multimodal approaches centered on genetic molecular data inadequately leveraged data structure and ignored the involvement of genes in the pathway or biological processes, thereby hampering interpretability of association models. In this study, a novel joint similarity nonnegative matrix factorization (JSNMF) model based on data-driven idea was proposed by adding pathway scoring data based on utilizing pathological images of tumor, gene expression data. The similarity network fusion model was applied to calculate the fusion matrices of the three-modality data with tumor recurrence as the label. Additionally, the prior information was calculated using the principal component analysis method, which was then applied to the joint nonnegative matrix factorization model with network regularization constraints. The solving efficiency of JSNMF model was enhanced by incorporating sparse orthogonality constraints on objective function. Experimental results demonstrate that incorporating prior knowledge enhances the search efficiency for joint patterns across multimodal data. The model identified recurrence-related common modules, including cellular features, genes, and pathways. Bioinformatics analysis indicated that the model can identify potential biomarkers associated with immune cell infiltration levels for recurrence diagnosis. Furthermore, the proposed method provides a new perspective for mining task-specific associations in multimodal data. This study also improves understanding of association patterns among genetic molecular features linked to tumor recurrence.

## Introduction

Tumors, a heterogeneous group of diseases characterized by invasive growth and tissue destruction, primarily rely on surgical resection as curative intervention, in contrast to radiotherapy which typically serves adjuvant roles [1–4]. Postoperative recurrence across cancer types remains a critical clinical challenge, often necessitating repeated interventions, imposing substantial economic burdens, and worsening patient outcomes [5–7]. Identifying key recurrence determinants and implementing timely interventions are urgent priorities in oncology, as they are essential for tailoring surveillance and adjuvant strategies to enhance patient prognosis.

Investigating the pathological features or molecular biological mechanisms associated with tumor recurrence is a fundamental approach to comprehend the progression of the tumor recurrence process [8]. Zhang *et al.* [9] conducted a statistical analysis of 171 synovial sarcoma cases to investigate the risk factors related to local recurrence. This study revealed that larger initial tumors, positive resection margins, marginal resection, lack of adjuvant therapy, multiple recurrences, were crucial factors linked to higher rates of local recurrence. Later, the samples who underwent more thorough tumor resection initially demonstrated a more positive prognosis [10]. Concurrently, multiple studies have explored molecular correlates of tumor recurrence. Schelbert *et al.* [11] conducted morphological, immunohistochemical, and molecular analyses of follicular dendritic cell sarcoma, revealing that the expression of adhesion molecule L1CAM can serve as a marker for diagnosing sarcoma recurrence. Wang *et al.* [12] demonstrated that ZNF703 in combination with STK11 enables accurate prediction of recurrence in triple-negative breast cancer. Besides, Watanabe *et al.* [13] revealed that Prostate-Specific Membrane Antigen (PSMA)-positive tumor vessels in renal cell carcinoma exhibit high angiogenic potential, serving as both therapeutic targets and recurrence predictors of kidney renal clear cell carcinoma. Recently, Ye *et al*. [14] investigated the dual function of necrotic cells in both the immune microenvironment and tumor progression, while also highlighting the potential use of necrotic cells as a novel target in sarcoma therapy. However, previous research has primarily focused on the pathological or genetic molecular characteristics of tumor, overlooking the connection between genetic molecular features and pathological tissues. Insights into how genetic molecular features impact cellular features in pathological tissues have been also neglected. Thus, the investigation of the effects of genetic molecular processes on cellular-level characteristics within the tumor microenvironment is a novel insight to increase awareness of the developmental pattern of recurrence-related molecular biological traits in tumor.

Multimodal data fusion algorithms have been extensively proposed to explore associations among modal data of tumor and to comprehend patterns of micro to macro feature development. The joint nonnegative matrix factorization (JNMF) model projects data from multiple perspectives into the same space and extracts associations between them through shared feature coefficients [15]. Recent studies have confirmed the dependability of JNMF models cross several application scenarios, including cancer diagnosis [16], regulatory networks [17], biomarker mining [18], and endogenous competitive network construction [19]. Later, the constrained JNMF framework was proposed for sarcoma-related multimodal data association studies to identify the image genetics association patterns linked to sarcoma lung metastasis [20]. This was followed by the discovery of genetic variation features related to cellular features [21]. Nonetheless, current studies are delimited in two ways. The existing studies have primarily focused on the correlation between individual single genetic molecules and image features. However, genetic molecules (such as genes) in real-world situations are influenced by environmental and other factors, and typically perform distinct biological functions as part of pathways [22, 23]. This hampers the interpretation of association results. Besides, current constrained JNMF techniques frequently construct the network regularization constraint using data associations from existing databases (e.g. regulatory relationships and protein interactions) and adjacency matrices generated via simple Pearson correlation calculations. Unfortunately, these approaches failed to account for the biological relationships among the data themselves. These techniques may result in a significant number of false-positive correlations, hindering the detection of potential correlation patterns in the data. Therefore, it is possible that the investigation of association patterns among multimodal data that are consistent with the biological context could be restricted.

**Figure 1:**
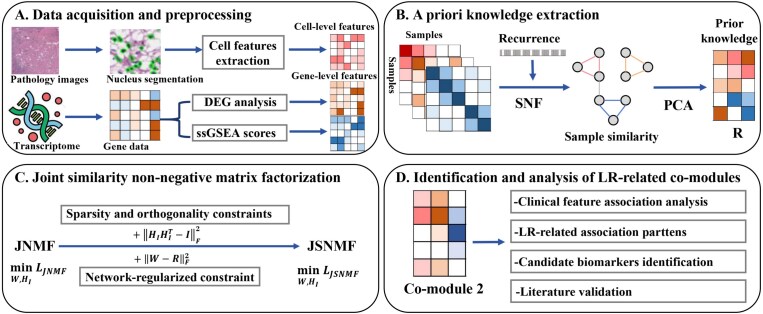
Workflow scheme illustrating (A) extraction of cell-level and gene-level features, (B) prior knowledge calculation from sample similarity matrices via SNF and PCA models, (C) derivation of the JSNMF model with prior knowledge-based network regularization and sparse-orthogonality constraints, and (D) identification and analysis of local recurrence-related common modules by bioinformatics.

In this study, a joint similarity nonnegative matrix factorization (JSNMF) model driven by a specific task was proposed to include the enriched pathway information of tumor samples for the first time. The method was to delve into the association patterns between pathological images and transcriptomic data from pathways or biological processes. The recurrent event was initially employed as a task to calculate the fusion matrix of three raw data based on the similarity network fusion (SNF) model. Subsequently, a prior knowledge matrix was extracted by implementing the principal component analysis (PCA) algorithm and was then integrated into the objective function to restrict the updated outcomes of the base matrices. The framework of the JSNMF method is shown in Fig. 1.

The innovations of this study can be summarized into three aspects:

- Proposing, for the first time, the concept of tri-modal data fusion that encompasses pathological images, gene expression, and pathway processes, which provides a novel perspective for revealing tumor recurrence mechanisms and identifying associations of tumor recurrence modules.
- Proposing a method that fuses tri-modal data based on SNF technology and integrates prior knowledge acquired via PCA, thereby establishing a new semi-supervised learning paradigm for matrix factorization.
- Developing a strategy that identifies co-module based on recurrence labels to mine the association patterns of tumor cross-modal recurrence modules, which offers an effective technical approach for the screening and identification of tumor biomarkers.

## Materials and methods

### Joint nonnegative matrix factorization model

Assume ${X}_{I} (I=1,2,3)$ represents the original data, where ${X}_{1}$ represents the whole slide imaging (WSI) feature matrix, ${X}_{2}$ represents the gene expression matrix, ${X}_{3}$ represents the pathway score matrix, factorize the original matrix ${X}_{I}$ into the a basis matrix $W$ and three coefficient matrices $H_{I}$:


(1) \begin{align*}& {X}_{I}\approx{W}{H}_{I},\ subject\ to\ {W}\geq0,\ {H}_{I}\geq0,\ I=1,2,3.\end{align*}

The objective function of nonnegative matrix factorization model is defined as:


(2) \begin{align*}& f({W},{H}_{I})=\sum_{I=1}^{3}\Vert X_{I}-WH_{I} \Vert_{F}^{2},\ W\geq0,\ H_{I}\geq0.\end{align*}

Lee and Seung [24] proposed a multiplicative iterative algorithm based on minimizing the Euclidean error function, which can redescribe the objective function $f({W},{H}_{I})$ as:


(3) \begin{align*} f(W, H_{I}) =& \sum_{I=1}^{3} \Big( \mathrm{Tr}\big(X_{I} X_{I}^{T}\big) - 2\, \mathrm{Tr}\big(X_{I} H_{I}^{T} W^{T}\big)\nonumber \\ & + \mathrm{Tr}\big(W H_{I} H_{I}^{T} W^{T}\big) \Big). \end{align*}

Let $\psi _{ij}$ and $\varphi _{ij}$ be Lagrange multipliers for ${W}_{ij}\geq 0$ and ${H}_{ij}\geq 0$, respectively. The objective function was defined as,


(4) \begin{align*} L(W, H_{I}) =\ & f(W, H_{I}) + \mathrm{Tr}(\Psi W) + \sum_{I=1}^{3} \mathrm{Tr}(\Phi H_{I}),\nonumber \\ & \Psi = [\psi_{ij}], \quad \Phi = [\varphi_{ij}]. \end{align*}

$L(W, H_{I})$ takes the partial derivative of $W$ and ${H}_{I}$, respectively,


(5) \begin{align*}& \left\{\begin{aligned} &\frac{\partial L}{\partial{W}}=\sum\nolimits_{I=1}^{3}\big(-2{X}_{I}{H}_{I}^{T}+2{W}{H}_{I}{H}_{I}^{T}\big)+{\Psi}\\ &\frac{\partial L}{\partial{H}_{I}}=-2{W}^{T}{X}_{I}+2{W}^{T}{W}{H}_{I}+{\Phi}. \end{aligned}\right.\end{align*}

Based on the Karush–Kuhn–Tucker (KKT) condition, ${W}_{ij} $ and $({H}_{I})_{ij}$ were calculated as follows:


(6) \begin{align*}& \left\{\begin{aligned} &-\sum\nolimits_{I=1}^{3}{({X}_{I}{H}_{I}^{T})}_{ij}{W}_{ij}+\sum\nolimits_{I=1}^{3}\big({W}{H}_{I}{H}_{I}^{T}\big)_{ij}{W}_{ij}=0\\ &{-\big({W}^{T}{X}_{I}\big)}_{ij}{{H}_{I}}_{ij}+\big({W}^{T}{W}{H}_{I}\big)_{ij}{{H}_{I}}_{ij}=0, \end{aligned}\right.\end{align*}

The updated rules for $W$ and $H_{I}$ are expressed as,


(7) \begin{align*}& \left\{\begin{aligned} &{W}_{ij}\gets{W}_{ij}\frac{\sum_{I=1}^{3}\big({X}_{I}{H}_{I}^{T}\big)_{ij}}{\sum_{I=1}^{3}({W}{H}_{I}{H}_{I}^{T})_{ij}}\\ &{{H}_{I}}_{ij}\gets{{H}_{I}}_{ij}\frac{\big({W}^{T}{X}_{I}\big)_{ij}}{\big({W}^{T}{W}{H}_{I}\big)_{ij}}. \end{aligned}\right.\end{align*}

### A prior knowledge extraction based on similarity network fusion

The prior information $R$ based on the SNF algorithm was extracted to make full use of the existing information of the original data. Then the constraints on $R$ were regularized into the objective function of the JNMF model, where a semi-supervised learning model was applied to reduce the randomness of the results and the time of searching data association patterns.

Assume that there are $n$ samples and $m$ data types, the data matrix is ${X}_{n \times q_{m}}^{(m)}$, where $n \times q_{m}$ denotes the size of data type $m$. Then the pair-wise distance (or similarity) matrix for each data matrix can be represented as:


(8) \begin{align*}& dist(m)=\Vert x_{i}-x_{j} \Vert_{n\times n} \in R_{+}^{n\times n},\end{align*}

where $R_{+}$ denotes the set of nonnegative real numbers.

Then, define the $S^{(m)}$ is normalized from the similarity matrix $dist(m)$, the k-nearest neighbor affinity matrix is defined as:


(9) \begin{align*}& {A}_{ij}^{(m)}=\left\{ \begin{aligned} &(1-\varepsilon)\frac{S_{ij}^{(m)}}{\sum_{j\in N_{k}(i)} S_{ij}^{(m)}}\\ &\varepsilon\frac{S_{ij}^{(m)}}{\sum_{j\notin N_{k}(i)} S_{ij}^{(m)}} \end{aligned} \right.\end{align*}

where $N_{k}(i)$ denotes the indexes of $k$ nearest neighbors of sample $i$. $\varepsilon $ is a small number.

Next, the fused affinity matrix can be calculated as:


(10) \begin{align*}& {A}=\ \sum_{1}^{m}{w_{m}\bullet}{A}^{(m)},\ \ \ \ \sum_{1}^{m}{w_{m}=1\ \cap\ w_{m}\geq0}.\end{align*}

Finally, spectral clustering on fused affinity matrix $A$ can be performed to verify the results of fusing multiple data types by setting the cluster number $C$. In this study, the sample label of recurrence was applied to be the output of the affinity network fusion. The cluster number $C$ is set as 2.

The study aims to explore the data association related to tumor recurrence. Thus, the matrix ${R}$ of a prior knowledge from the fused affinity matrix $A_{n\times n}$ was calculated by the PCA algorithm, defined as $R=PCA(A, K)$.

### Joint similarity nonnegative matrix factorization model

After extracting prior knowledge $R$, the regularization constraint was denoted as $\Vert W-R\Vert _{F}^{2}$. The NMF algorithm is sensitive to data quality, and its objective function has the drawback of failing to ensure the sparsity of the original data, making it necessary to control the sparsity of $W$ or $H$. Previous studies have shown that imposing orthogonal constraints on the objective function can effectively render the coefficient matrix $H$ sparse [20, 25]. The objective function can be redefined as:


(11) \begin{align*} f(W, H_{I}) = &\min \Bigg[ \sum_{I=1}^{3} \Big( \left\| X_{I} - W H_{I} \right\|_{F}^{2} + \beta \left\| H_{I} H_{I}^{T} - I \right\|_{F}^{2} \Big)\nonumber \\ & + \alpha \left\| W - R \right\|_{F}^{2} \Bigg], \end{align*}

where $\alpha $ is to govern the similarity between $W$ and $R$, $\beta $ is a hyperparameter to control the orthogonality of $H_{I}$.

Let $\psi _{ij}$ and $\varphi _{ij}$ be Lagrange multipliers for ${W}_{ij}\geq 0$ and $({H}_{I})_{ij}\geq 0$, respectively. The objective function is indicated as:


(12) \begin{align*} L(W, H_{I}) =\ & f(W, H_{I}) + \mathrm{Tr}\big(\Psi W^{T}\big) + \sum_{I=1}^{3} \mathrm{Tr}\big(\Phi H_{I}^{T}\big),\nonumber \\ & \Psi = [\psi_{ij}], \quad \Phi = [\varphi_{ij}]. \end{align*}

$L(W, H_{I})$ can be obtained by taking partial derivatives of $W$ and $H_{I}$ separately:


(13) \begin{align*}& \left\{ \begin{aligned} &\frac{\partial L}{\partial W}=\sum\nolimits_{I=1}^{3}\big(-2X_{I}H_{I}^{T}+2WH_{I}H_{I}^{T}\big)+2\alpha W- 2\alpha R +\Psi\\ &\frac{\partial L}{\partial{H}_{I}}=-2{W}^{T}{X}_{I}+2{W}^{T}{W}{H}_{I}+2\beta\big[\big({H}_{I}{H}_{I}^{T}-{E}\big){H}_{I}\big]+ \Phi, \end{aligned} \right.\end{align*}

where the elements of $E$ are all 1. Based on the KKT condition, ${W}_{ij}$ and ${H}_{ij}$ can be obtained as follows:


(14) \begin{align*}& \left\{ \begin{aligned} & -\left( \sum\nolimits_{I=1}^{3} X_{I} H_{I}^{T} + \alpha R \right)_{ij} W_{ij} + \left( \sum\nolimits_{I=1}^{3} W H_{I} H_{I}^{T} \right)_{ij} W_{ij} \\ &\quad +\ (\alpha W)_{ij} W_{ij} = 0 \\ & - (W^{T} X_{I})_{ij} H_{Iij} + \left[ W^{T} W H_{I} \right]_{ij} H_{Iij} \\ &\quad +\ \left[ \beta(H_{I} H_{I}^{T} - E) H_{I} \right]_{ij} H_{Iij} = 0. \end{aligned} \right.\end{align*}

Ultimately, the update equation for $W$ and $H$ is formulated as,


(15) \begin{align*}& \left\{ \begin{aligned}&{W}_{ij}\gets{W}_{ij}\frac{{\left(\sum_{I=1}^{3}{{X}_{I}{H}_{I}^{T}+\alpha{R}}\right)}_{ij}}{\left(\sum_{{I}=\mathbf{1}}^{\mathbf{3}}{{W}{H}_{I}{H}_{I}^{T}}+\alpha{W}\right)_{ij}}\\&{{H}_{I}}_{ij}\gets{{H}_{I}}_{ij}\frac{\big({W}^{T}{X}_{I}\big)_{ij}}{\big({W}^{T}{WH_{I}}+\beta\big({H}_{I}{H}_{I}^{T}-{E}\big){H}_{I}\big)_{ij}}.\\ \end{aligned} \right.\end{align*}

$W$ and $H$ are updated on an ongoing basis to meet the convergence rule, i.e. the relative error of reaching a specified value or reaching a defined iteration number. The $W$ and $H$ matrices are initialized by the nonnegative double singular value decomposition algorithm to guarantee the reproducibility of results.

### Convergence proof of the algorithm

Theorem 1.1. Under the above update rule Equation (15), the objective function Equation (11) is not increased. The objective function remains unchanged under these updates if and only if $W$ and $H_{I} (I=1, 2, 3)$ are at the stationary point of the objective function.

The standard proof of convergence for NMF can be extended to prove this theorem. It is known that the lower bound of the objective function Equation (11) is zero. Regarding the upper limit, it is necessary to demonstrate that the objective function remains constant when using the multiplication update formula and remains static at the local minimum. The characteristics of auxiliary functions were used to achieve this proof by Lee and Seung [26]. To simplify the proof, the objective function does not increase under the update rule of $H_{I} (I=1, 2, 3)$ were proven, and the objective function does not increase under the update rule of $W$, which can be proven in the same way.

Definition 1.
$G(h,h^{\prime })$ is an auxiliary function of $F(h)$ when it satisfies the following conditions.

(16) \begin{align*}& G(h,h^{\prime})\geq F(h), G(h,h)=F(h).\end{align*}

Lemma 1. If $G$ is an auxiliary function of $F$, then $F$ does not increase under the following update rule.

(17) \begin{align*}& h^{(t+1)}=argmin \ G\big(h,h^{t}\big).\end{align*}

$F_{ab}$ was used to represent the related elements in the objective function, where ${H_{I}}_{ab}$ is the element in $H_{I}$. $F_{ab}$ and its first and second derivatives for $H_{I}$ are as follows:


(18) \begin{align*} F_{ab}(H_{I}) = & \left[ \sum_{I=1}^{3} \left( \left\| X_{I} - W H_{I} \right\|_{F}^{2} + \beta \left\| H_{I} H_{I}^{T} - E \right\|_{F}^{2} \right) \right]_{ab} \nonumber\\ & + \left[ \alpha \left\| W - R \right\|_{F}^{2} \right]_{ab}, \end{align*}

(19) \begin{align*} F^{\prime}_{ab}(H_{I}) = \left( \frac{\partial F}{\partial H_{I}} \right)_{ab} =\ & \left( -2 W^{T} X_{I} + 2 W^{T} W H_{I} \right. \nonumber\\ & \left. +\ 2 \beta ( H_{I} H_{I}^{T} - E ) H_{I} \right)_{ab}, \end{align*}

(20) \begin{align*} F_{ab}^{\prime\prime}(H_{I}) =\ & \left( \frac{\partial^{2} F}{\partial H_{I}^{2}} \right)_{ab} = \left[ 2 W^{T} W + 2 \beta ( H_{I} H_{I}^{T} - E ) \right]_{aa}. \end{align*}

Since updates are element-by-element in nature, it is sufficient to prove that each $F_{ab}({H}_{I})$ is not increased under the $H_{I}$ update rule.

Lemma 2. If $K_{ab} (H_{I})$ is a diagonal matrix

(21) \begin{align*}& K_{ab}\left( H_{I}^{(t)} \right) = \frac{{\delta_{ab}\left( {F^{\prime\prime}_{ab}\left( H_{I}^{(t)} \right)} \right)}_{ab}}{H_{Iab}^{(t)}}.\end{align*}

then, $F_{ab}(H_{I})$ at $H_{ab}^{t}$ can be changed to the following form:


(22) \begin{align*} G_{ab}\big(H_{I}, H_{I}^{(t)}\big) =&\ F_{ab}\big(H_{I}^{(t)}\big) + F_{ab}^\prime\big(H_{I}\big)\big(H_{Iab} - H_{Iab}^{(t)}\big)\nonumber \\ &+ \big[W^{T}W + \beta\big(H_{I} H_{I}^{T} - E\big)\big]_{ab}\big(H_{Iab} - H_{Iab}^{(t)}\big)^{2}. \end{align*}

Equation (22) is the function overall ($H_{I}$) auxiliary function.

Proof. The Taylor second-order expansion of $F_{ab}({H}_{I})$ was performed as follows:

(23) \begin{align*} F_{ab}(H_{I}) =\ & F_{ab}^{(t)}(H_{I})_{ab} + \left( \frac{\partial F}{\partial H_{I}} \right)_{ab} \cdot (H_{Iab} - H_{Iab}^{(t)})\nonumber \\ & + \left[ W^{T} W + \beta(H_{I} H_{I}^{T} - E) \right]_{ab} \cdot \big(H_{Iab} - H_{Iab}^{(t)}\big)^{2}. \end{align*}

Comparing auxiliary function and Equation (21), $G_{ab}({H}_{I},{H}_{I}) =F_{ab}({H}_{I})$ is obvious. So only $G_{ab}{({H}}_{I},{H}_{I}^{({t})})\geq \ F_{ab}({H}_{I}^{({t})})$ was proved.


(24) \begin{align*} \left( [W^{T} W + \beta(H_{I} H_{I}^{T} - I)] H_{I} \right)_{ab} =& \sum_{k} \left[ W^{T} W + \beta\big(H_{I} H_{I}^{T} - E\big) \right]_{ak} \cdot (H_{I})_{kb}\nonumber \\ &\geq (H_{I})_{ab} \cdot \left[ W^{T} W + \beta\big(H_{I} H_{I}^{T} - E\big) \right]. \end{align*}

Therefore, $G_{ab}{({H}}_{I},{H}_{I}^{({t})})\geq \ F_{ab}({H}_{I}^{({t})})$ was established.

(Proof of Theorem 1.1) Based on the auxiliary function, the following update rule was obtained:

(25) \begin{align*}& {{H}_{I}}_{ij}\gets{{H}_{I}}_{ij}\frac{({W}^{T}{X}_{I})_{ij}}{({W}^{T}{WH}+\beta({H}_{I}{H}_{I}^{T}-{E}){H}_{I})_{ij}}.\end{align*}

Depending on the property of the auxiliary function, $F_{ab}$ does not increase under this update rule.

## Experiments and results

The accurate identification of potential biomarkers for tumor recurrence diagnosis serves as a critical indicator for evaluating the performance of the JSNMF model. In this context, the present study conducts a series of bioinformatics analyses on three tumor datasets to validate capability of the JSNMF model in mining biomarkers associated with tumor recurrence.

### Data collection and preprocessing

Pathological images, transcriptome, and clinical data of tumor samples were downloaded from The Cancer Genome Atlas Program (TCGA) website (https://www.cancer.gov/). The pathway scores data were downloaded from the University of California Santa Cruz (UCSC) website (http://xena.ucsc.edu/). The study included three tumor types: sarcoma (SARC), triple-negative breast cancer (TNBC), and kidney renal clear cell carcinoma (KIRC). The specific information of the three tumor datasets is shown in Table 1.

**Table 1:** Details of the three datasets

Clinical parameters	SARC	TNBC	KIRC
Sample size	188	66	237
WSI size	150	150	150
Gene size	869	570	1509
Pathway size	1387	1387	1387
*Age (years)*			
Range	20–90	29–82	29–90
Median	62	53	61
*Gender*			
Female	100 (53.2%)	66 (100%)	76 (32.1%)
Male	88 (46.8%)	0 (0%)	161 (67.9%)
*Status*			
Alive	115 (61.2%)	57 (86.4%)	161 (67.9%)
Dead	73 (38.8%)	9 (13.6%)	76 (32.1%)
*Local recurrence*			
Yes	52 (27.7%)	8 (12.1%)	75 (31.6%)
No	136 (72.3%)	58 (87.9%)	162 (68.4%)

For pathology images, nucleus segmentation, cell-level feature extraction, and aggregation of cell-level features into patient-level features were performed on all WSI data by the processing procedures used in the previous studies [27]. Ten different cell-level features were extracted from each segmented nucleus. Then, a 10-binary histogram (denoted by bin1, bin2...bin10) and 5 statistical measures including mean, SD, skewness, kurtosis, and entropy were calculated for each cell-level feature to obtain 150 patient-level features. For transcriptome data, all samples were divided into local recurrence and nonrecurrence samples. Then the Wilcoxon test was applied to perform the differential expression analysis on all genes between the two groups. Finally, genes with $P$-values <.01 were selected for subsequent analysis (the threshold of TNBC dataset is $P$-value <.05). The ssGSEA scores of the pathways were calculated by using the Pathway Representation and Analysis by Direct Reference on Graphical Models. The SuperPathway structure includes 1387 constituent pathways from three pathway databases: NCI-PID, BioCarta, and Reactome.

### Parameter setting

**Figure 2:**
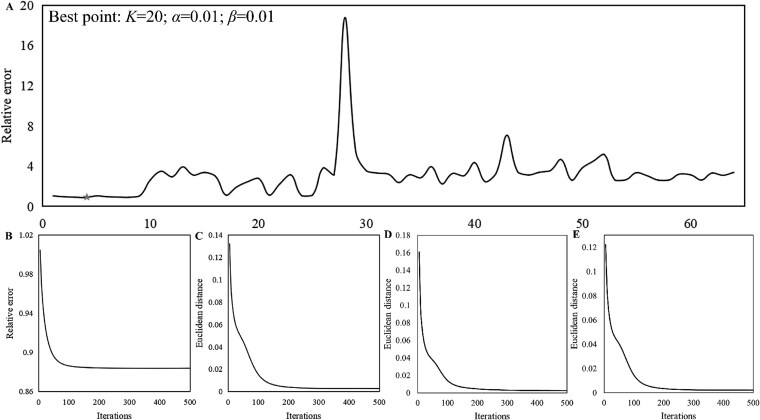
(A) the relative error of 64 combinations of three parameters in the SARC dataset, (B) the relative error of the model for parameter combination with $K = 20$, $\alpha $=0.01, and $\beta $=0.01, (C) Euclidean distance of $H_{1}$ for 500 iterations, (D) Euclidean distance of $H_{2}$ for 500 iterations, (E) Euclidean distance of $H_{3}$ for 500 iterations.

The hyperparameter selection determines the factorization performance of the model and the number of co-modules. Figure 2 displays the convergence effect of the model based on parameter selection. The relative error was set as the objective $\sum _{I=1}^{3}mean\left (\frac{mean\left (\left |X_{I}-WH_{I}\right |\right )}{mean\left (X_{I}\right )}\right )$ to construct the optimal model. It has been found that the selection of the number of components in NMF is generally an order of magnitude smaller than the sample size [26]. Thus, the value range of $K$ is set to $\{5,10,15,20\}$. According to previous studies [28], the value range of $\alpha $ or $\beta $ is set to $\{0.01,0.1,1,10\}$. The number of iterations of the model is set to 500 under 64 three hyperparameters-combinations.

As shown in Fig. 2, among 64 parameter combinations, the relative error of the objective function of the JSNMF model is minimized to 0.884 when $K$ = 20, $\alpha $ = 0.01, and $\beta $ = 0.01 in the SARC dataset. In addition, this study recorded the relative errors of TNBC and KIRC under different parameter combinations in Supplementary Fig. S1 and found that $K$ = 20, $\alpha $ = 0.01, and $\beta $ = 0.01 were still the optimal parameter combinations. Meanwhile, the Euclidean distance of matrices $H_{I}$ and the relative error tends to converge after the number of iterations reaches 500.

### Validation of the factorization performance of the joint similarity nonnegative matrix factorization model

**Figure 3:**
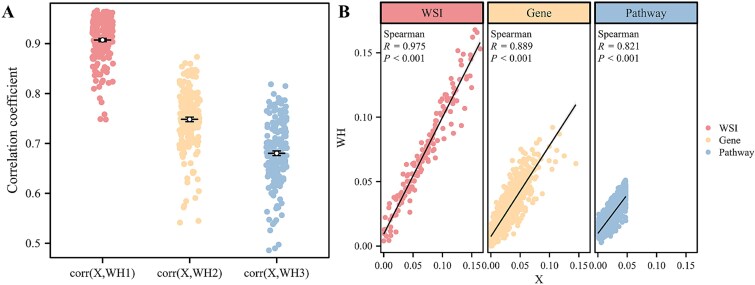
(A) a violin plot of Pearson correlation between $X$ and $WH$ in three types of SARC data, and (B) a scatterplot of the Pearson correlation between $X$ and $WH$ for the WSI, gene, and pathway in SARC sample 44.

The data correlation of each sample after matrix factorization is one of the methods to measure the performance of model factorization. In this study, the Pearson correlation coefficient of each sample data before and after factorization was calculated, then the overall factorization effect of data of three modes in all SARC samples is shown in Fig. 3A, while the model factorization effect of each SARC sample is shown in Fig. 3B. In addition, the factorization effect of TNBC and KIRC datasets is shown in Supplementary Fig. S2.

As can be seen in Fig. 3, for all three types of SARC data, the average correlation between the raw data and the Factorized matrix product across all samples shows a significant correlation with WSI: 0.907 (95% CI: 0.889, 0.936), Gene: 0.748 (95% CI: 0.685, 0.810), and Pathway: 0.680 (95% CI: 0.601, 0.754). The performance in a single sample (e.g. sample 44) shows a significantly high correlation, indicating a good factorization of the model.

### Comparison with other methods

Based on the joint nonnegative matrix model, this study introduces orthogonality constraints, and network regularization constraint that are constructed based on the a prior information of SNF. To explore the effectiveness of introducing constraints, this study compares the model performance of JSNMF, OC-JNMF (JSNMF without SNF model), SNF-JNMF (JSNMF without orthogonality constraints), JNMF, SVD_JNMF [29], DSRJNMF [30], JCB-SNMF [31], OSJNMF-C [32], scMNMF [33], MDJNMF [21], SPID-MDJNMF [34] in five evaluation metrics. These metrics comprise the time cost of model factorization, the relative error and Euclidean distance between the original data $X_{I}$ and the product $WH_{I}$ of the Factorized base matrix $W$ and coefficient matrix $H_{I}$, the average correlation between $X_{I}$ and the reconstructed data (denoted as $XX_{I}$), which is the product of $WH_{I}$, calculated as $\text{Cor}(X_{I}, XX_{I})$, and the overlapping ratio $O$, defined as $O = m/n$, where $m$ denotes the number of intersection between the enriched pathway for $H_{2}$-based significant genes and the H3-based significant pathways in the co-module, $n$ denotes the enriched pathway for $H_{2}$-based significant genes.

The equipment used in this study was an Intel CoRE i5-13500HX processor, Matlab R2023a. The study compares the factorization effects of different algorithms on three tumor datasets. Tables 2–4 show the factorization of SARC, TNBC, and KIRC datasets respectively. In the study, values in bold represent the best performance under the corresponding metric, and underlined values indicate the second-best performance under the metric.

**Table 2:** Comparison of matrix factorization effects of 11 algorithms in SARC dataset

Methods	Time	Relative error	Euclidean distances	$Cor(X_{1}, XX_{1})$	$Cor(X_{2}, XX_{2})$	$Cor(X_{3}, XX_{3})$	Overlapping ratio		
							NCI-PID	Biocarta	Reactome
SVD-JNMF	**1.040**	0.959	77.220	0.873	0.714	0.618	0.500	0.330	0.180
DSRJNMF	12.681	0.999	82.745	0.848	0.696	0.594	0.430	0.120	0.180
JCB-SNMF	1.999	0.976	82.794	0.863	0.711	0.611	0.270	0.120	0.170
OSJNMF-C	13.424	0.999	82.934	0.847	0.696	0.594	0.200	0.250	0.200
scMNMF	9.463	1.020	86.488	0.823	0.694	0.567	0.250	0.500	0.210
MDJNMF	2.792	0.911	71.029	0.895	0.732	0.660	0.330	0.180	0.100
SPID-MDJNMF	2.772	0.952	76.800	0.878	0.721	0.644	0.200	0.080	0.250
JNMF	1.435	0.935	74.091	0.883	0.722	0.643	0.330	0.330	0.180
SNF-JNMF	1.456	0.919	71.652	0.892	0.731	0.653	0.430	0.290	0.090
OC-JNMF	1.490	0.920	71.434	0.892	0.733	0.661	0.600	0.400	0.200
JSNMF	1.438	**0.884**	**66.504**	**0.907**	**0.748**	**0.680**	**0.800**	**0.570**	**0.280**

**Table 3:** Comparison of matrix factorization effects of 11 algorithms in TNBC dataset

Methods	Time	Relative error	Euclidean distances	$Cor(X_{1}, XX_{1})$	$Cor(X_{2}, XX_{2})$	$Cor(X_{3}, XX_{3})$	Overlapping ratio		
							NCI-PID	Biocarta	Reactome
SVD-JNMF	1.278	0.628	8.021	0.994	0.972	0.650	0.43	0.33	0.20
DSRJNMF	7.674	0.663	9.151	0.992	0.967	0.615	0.33	0.25	0.11
JCB-SNMF	1.045	0.675	8.705	0.992	0.970	0.642	0.40	0.33	0.24
OSJNMF-C	7.545	0.669	9.294	0.991	0.967	0.608	0.33	0.25	0.11
scMNMF	9.690	0.851	12.870	0.988	0.952	0.445	0.33	0.25	0.25
MDJNMF	1.612	0.642	8.385	0.994	0.972	0.623	0.43	0.33	0.21
SPID-MDJNMF	1.969	0.599	6.964	0.995	0.979	0.662	0.50	0.33	0.27
JNMF	0.568	0.576	6.475	**0.996**	0.979	0.692	0.40	0.33	0.28
SNF-JNMF	**0.546**	0.588	6.703	0.995	0.978	0.687	0.60	0.33	0.29
OC-JNMF	0.591	0.605	7.248	0.993	0.976	0.694	0.44	0.22	**0.34**
JSNMF	0.572	**0.559**	**6.016**	0.995	**0.981**	**0.720**	**0.60**	**0.50**	0.30

**Table 4:** Comparison of matrix factorization effects of 11 algorithms in KIRC dataset

Methods	Time	Relative error	Euclidean distances	$Cor(X_{1}, XX_{1})$	$Cor(X_{2}, XX_{2})$	$Cor(X_{3}, XX_{3})$	Overlapping ratio		
							NCI-PID	Biocarta	Reactome
SVD-JNMF	**1.618**	0.727	49.429	0.990	0.921	0.795	**0.67**	0.40	0.32
DSRJNMF	17.268	0.759	55.631	0.987	0.909	0.785	0.25	0.40	0.32
JCB-SNMF	2.081	0.751	51.832	0.987	0.918	0.798	0.33	0.40	0.31
OSJNMF-C	16.721	0.760	55.984	0.987	0.908	0.787	0.33	0.40	0.29
scMNMF	12.889	0.728	49.091	0.989	0.920	0.798	0.33	0.40	0.31
MDJNMF	3.786	0.727	50.067	0.990	0.919	0.796	0.25	0.40	**0.35**
SPID-MDJNMF	4.854	0.712	44.946	0.990	0.931	0.801	0.25	0.50	0.33
JNMF	1.650	0.698	42.912	0.991	0.935	0.806	0.33	0.40	0.29
SNF-JNMF	1.957	0.705	44.102	0.991	0.933	0.804	0.33	0.40	0.26
OC-JNMF	1.939	0.700	44.010	0.991	0.932	0.808	0.33	0.50	0.33
JSNMF	1.878	**0.669**	**38.254**	**0.993**	**0.943**	**0.811**	0.36	**0.60**	0.33

From Tables 2–4, it can be seen that JSNMF performs better in factorization and finds more biologically meaningful co-modules between the data, mainly in the sense that the pathways enriched by the featured genes have a higher overlap with the original featured pathways. This suggests that the inclusion of a prior knowledge clusters more elements with potentially related information. However, it is clear that the addition of constraints increases the complexity of the model, but the actual operation efficiency is still in the top three of all algorithms. Furthermore, this study also presents the performance comparison of JSNMF with three deep learning methods, including SGEGCAE [35], MOGLAM [36], and OmiEmbed [37], in Supplementary Table S1. The results still demonstrate that JSNMF exhibits excellent matrix reconstruction capability and operational efficiency.

This study computed the time complexity and space complexity of JSNMF and baseline algorithms to compare their running speeds, with detailed results presented in Supplementary Table S2. The derivation process for time complexity and space complexity of JSNMF is provided in Supplementary File S1. Comparison of time and space complexities reveals that JSNMF exhibits lower complexity than other algorithms. This enables JSNMF to avoid occupying excessive memory and computation time during operation, thereby contributing to its superior running speed over other comparative algorithms.

## Bioinformatics analyses

### Clinical feature association based on basis matrix

**Figure 4:**
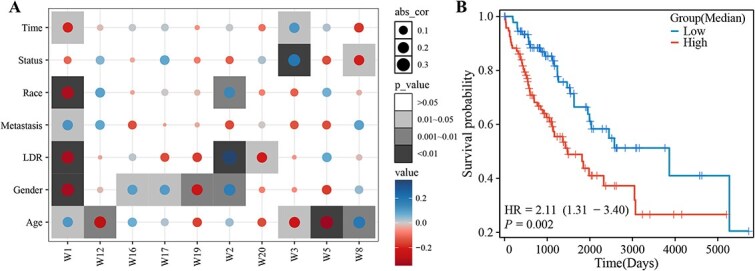
(A) the Pearson correlation between all $W$ matrices and seven clinical indices, including Time (overall survival time), Status (overall status), Race, Metastasis, local disease recurrence (LDR), Gender, and Age, and (B) KM survival curves based on the risk score. High, high-risk; Low, low-risk.

The base matrix $W$ represents the common features of three types of data. This study takes SARC dataset as the research object, the Pearson correlation coefficient between the values of matrix $W$ and seven clinical indices was calculated to explore the clinical significance of common modules, as shown in Fig. 4A. Proportional risk hypothesis testing was performed using the “survival” R package, and univariate cox regression analyses was performed on all $W$ vectors, and the vectors of $P$-value <.05 were then selected into the multivariate cox analysis. The risk score based on all $W$ vectors was calculated by multivariate analysis. The model was represented as $riskscore=\ -1.544\times W_{3}+4.242\times W_{8}+5.128\times W_{11}$. The Kaplan–Meier (KM) survival analysis was performed on high- and low-risk groups divided by the risk score in Fig. 4B.

From Fig. 4A, 10 of 20 $W$ vectors are significantly related to clinical index, where $W_{1}$ and $W_{20}$ are positively correlated with local disease recurrence, and $W_{2}$ are negatively correlated with local disease recurrence. The prognosis is better in samples with higher risk scores, as shown in Fig. 4B. This evidence suggests that the JSNMF algorithm is able to find common features that correlate with clinical metrics, particularly common modules that are significantly associated with local disease recurrence.

To verify that tri-modal data fusion (WSI-gene-pathway) offers more explicit biological interpretability than bimodal data fusion, this study designed an ablation experiment at the data level. Specifically, based on the Pearson correlation coefficient, we analyzed the correlations between the computed $W$ matrices (from different modality combinations: WSI-gene-pathway, WSI-gene, WSI-pathway, and gene-pathway) and seven clinical indicators, with the results provided in Supplementary Fig. S3. Results showed that across the four aforementioned modality combinations, $W_{1}$ was consistently the co-module associated with the largest number of clinical indicators. Thus, $W_{1}$ was selected as the focus of the ablation experiment. Comparative analysis revealed that the $W_{1}$ matrix derived from WSI-gene-pathway tri-modal data fusion exhibited more significant correlations with clinical indicators. This indicates that tri-modal data fusion can effectively enhance capability of the model to mine biological information.

### Data association analysis of common module related to sarcoma recurrence

**Figure 5:**
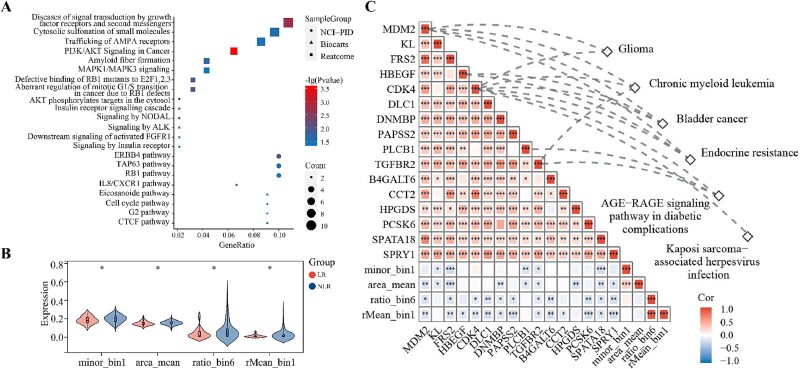
(A) Pathway enrichment results for genes in co-module 2. Group denotes the mapped pathways, including Biocarts, NCI-PID, and Reatcome pathways. The size of the circle denotes the count of genes in the pathway. (B) Expression of significant four cell features in local recurrence (LR) and No local recurrence (NLR). ^*^denotes $P<0.05$. (C) The relationship of three types of data. Diamonds indicate the description of the enriched KEGG pathway. Dashed lines indicate that the gene is involved in the pathway.

To identify the co-module most strongly associated with local disease recurrence, Pearson’s correlation coefficient was employed. The analysis focused on the SARC dataset in this section, where co-module 2 was specifically utilized to explore associations between cellular features, genes, and pathways. The “ClusterProfile” R package [38] was applied to perform pathway enrichment analysis on all genes in co-module 2. Then, the overlapping pathways between enriched pathways and significant H3-based pathways were shown in Fig. 5A. Four significant WSI features between local disease recurrence and control group were identified by independent samples Mann–Whitney test, as shown in Fig. 5B. KEGG pathway enriched analysis was performed on all genes. The Spearman correlation coefficient between four cell features and genes was calculated, and genes significantly associated with at least one image feature (significant $P$-value <.05) were obtained in Fig. 5C.

There are four overlapped NCI-PID pathways, four overlapped Biocarts pathways, and over ten overlapped Reatcome pathways in Fig. 5A. The overlapped pathways in co-module 2 are mainly tumor-related pathways, such as PI3K/AKT signaling in cancer, MAPK1/MAPK3 signaling and ERBB4 pathway, etc. In Fig. 5B, there were four cellular features that were significantly different in the local recurrence and control group. Figure 5C indicates primarily significant positive correlations among genes, while significant negative correlations were observed between genes and cellular features. These results suggest a higher level of consistency in the expression level of the same data within the common module. These genes are primarily involved in the sarcoma-associated KEGG pathways, such as Glioma, Chronic myeloid leukemia, etc.

Furthermore, we performed Gene Ontology analysis on the highly expressed genes in co-module 2 to explore core capability of JSNMF of mining biological significance and potential regulatory mechanisms, with the analysis results provided in Supplementary Fig. S4. Notably, existing studies have confirmed that autophagosomes exert a significant impact on the sarcoma fusion process [39]. Additionally, nuclear envelope collapse induces micronucleus damage, which in turn impairs genomic stability and triggers carcinogenic effects [40]; meanwhile, the activation pathway of sarcoma oncogenic growth factors is regulated by phosphatidylinositol 3–kinase (PI3K) [41]. These results indicate that JSNMF can reveal potential tumor pathogenic mechanisms by mining relevant genes.

The study performed mediation analysis on the differentially expressed genes, pathways, and pathological image features mined by JSNMF, for the purpose of further investigating the associations among different modalities. Since pathways are synergistically composed of multiple genes, and abnormal pathway activity directly regulates the biological behavior of tumor cells which regulatory effect can be visualized via pathological image features [42]. Therefore, this study employed a mediation analysis sequence of “gene-pathway-pathological image.” Specifically, this sequence confirms that JSNMF possesses significant cross-modal data association capability.

This section focused on an in-depth analysis of FRS2—a differentially expressed gene within co-module 2 mined by JSNMF. Specifically, we evaluated the average causal mediated effect (ACME), average direct effect (ADE) on changes in cellular features, total effect, and proportion mediated of this gene on cellular features via pathways. Additionally, the statistical significance of these effect relationships was analyzed using the “mediation” R package. Table 5 shows that FRS2 exerted significant indirect effects on three pathological image features which reflect cellular features via three pathways. This further confirms that JSNMF can mine associations between multimodal data through co-modules.

**Table 5:** Mediation analysis of the FRS2-mediated effect pathways

FRS2 mediating effect path	Regulatory relationship	Estimate	95% CI	$P$ -value
Trafficking_of_AMPA_receptors$\rightarrow $ minor_bin1	ACME	$-3.953 \times 10^{-7}$	$-8.36 \times 10^{-7},\ -1.12 \times 10^{-7}$	$<2 \times 10^{-16}$
	ADE	$-7.60 \times 10^{-7}$	$-1.40 \times 10^{-6},\ -1.70 \times 10^{-7}$	$0.008$
	Total effect	$-1.155 \times 10^{-6}$	$-1.83 \times 10^{-6},\ -5.33 \times 10^{-7}$	$0.002$
	Proportion mediated	$34.22\%$	$12.55\%,\ 74.10\%$	$0.002$
Validated_transcriptional_targets_of_TAp63_isoforms $\rightarrow $ minor_bin1	ACME	$1.626 \times 10^{-7}$	$1.50 \times 10^{-8},\ 4.16 \times 10^{-7}$	$0.034$
	ADE	$-1.318 \times 10^{-6}$	$-2.08 \times 10^{-6},\ -6.71 \times 10^{-7}$	$<2 \times 10^{-16}$
	Total effect	$-1.155 \times 10^{-6}$	$-1.85 \times 10^{-6},\ -5.45 \times 10^{-7}$	$<2 \times 10^{-16}$
	Proportion mediated	$-14.08\%$	$-41.59\%,\ -1.28\%$	$0.034$
ctcf_first_multivalent_nuclear_factor$\rightarrow $ area_mean	ACME	$-2.016 \times 10^{-7}$	$-3.81 \times 10^{-7},\ -6.67 \times 10^{-8}$	$<2 \times 10^{-16}$
	ADE	$-2.681 \times 10^{-7}$	$-6.66 \times 10^{-7},\ 1.37 \times 10^{-7}$	$0.188$
	Total effect	$-4.697 \times 10^{-7}$	$-8.75 \times 10^{-7},\ -9.80 \times 10^{-8}$	$0.022$
	Proportion mediated	$42.93\%$	$9.12\%,\ 160.54\%$	$0.022$
ctcf_first_multivalent_nuclear_factor$\rightarrow $ minor_bin1	ACME	$-4.357 \times 10^{-7}$	$-7.74 \times 10^{-7},\ -1.60 \times 10^{-7}$	$0.002$
	ADE	$-7.195 \times 10^{-7}$	$-1.43 \times 10^{-6},\ -6.38 \times 10^{-8}$	$0.038$
	Total effect	$-1.155 \times 10^{-6}$	$-1.84 \times 10^{-6},\ -5.32 \times 10^{-7}$	$<2 \times 10^{-16}$
	Proportion mediated	$37.71\%$	$11.93\%,\ 88.42\%$	$0.002$

### Identification of candidate biomarkers from co-modules

**Figure 6:**
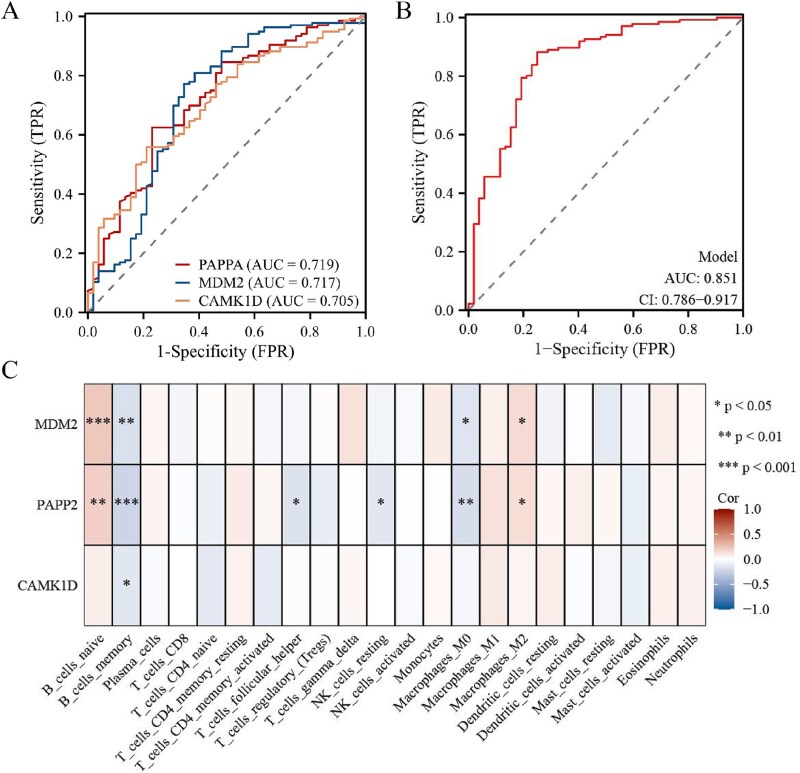
(A) ROC analysis of three genes. (B) ROC plot of three genes-combined model. (C) Correlation between the expression of 3 genes and the infiltration levels of 22 immune cells.

The analysis focused on the SARC dataset in this section. The receiver operating characteristic (ROC) analysis on the local recurrence and control groups was performed on the genes in Module 2 to investigate the biomarker mining potential of the co-modules obtained using the JSNMF algorithm in Fig. 6. Figure 6A presents the three genes with an area under the curve (AUC) >0.7. Furthermore, this study also presents the top three genes with the largest AUC mined via JSNMF in the TNBC and KIRC datasets, respectively, along with the predictive probability ROC curves of these three genes, which are shown in Supplementary Fig. S5. Subsequently, logistic regression analysis was performed based on these three genes to obtain predicted probabilities, and ROC analysis was conducted, as depicted in Fig. 6B. Additionally, the association between genes and immune cells was examined by calculating the infiltration levels of immune cells in the samples using CIBERSORT [38]. Figure 6C demonstrates the Pearson correlation coefficients of the three genes with the immune cells.

From Fig. 6A, the ROC curves showed good discriminative ability in the local recurrence group for PAPPA (AUC = 0.719, 95% CI: 0.637–0.802), MDM2 (AUC = 0.717, 95% CI: 0.625–0.809), and CAMK1D (AUC = 0.705, 95% CI: 0.625–0.785). Based on three genes, the predictor variable developed by logistic regression achieved an AUC of over 0.85, as shown in Fig. 6B. Figure 6C revealed that the expression levels of all genes consistently demonstrated a correlation with the infiltration level of immune cells. Specifically, the expression levels of three genes exhibited a negative correlation with the infiltration level of B cell memory. Moreover, the expression levels of MDM2 and PAPP2 were significantly negatively correlated with the infiltration level of Macrophages M0. Additionally, the expression levels of MDM2 and PAPP2 displayed a consistent positive correlation with the infiltration levels of B cells naive and Macrophages M2.

Based on the experimental results of JSNMF on the SARC dataset, this study further adopted the same method to evaluate the performance of seven mainstream multimodal data association algorithms and three ablation variants of JSNMF in predicting tumor recurrence across three datasets (SARC, TNBC, andKIRC), with performance evaluated by AUC values. Results are presented in Table 6, with optimal values bolded. Additionally, the predictive ROC curves of each algorithm on the aforementioned three datasets are provided in Supplementary Figs S6 (SARC), S7 (TNBC), and S8 (KIRC). Results demonstrate that JSNMF achieved the optimal AUC values in all three tumor datasets, confirming that its ability to mine tumor recurrence-related modules is significantly superior to that of other comparative methods.

**Table 6:** Comparison of the ability of 11 methods to predict tumor recurrence

Methods	SARC	TNBC	KIRC
SVD-JNMF	0.730	0.737	0.648
DSRJNMF	0.682	0.778	0.649
JCB-SNMF	0.762	0.812	0.681
OSJNMF-C	0.671	0.812	0.690
scMNMF	0.674	0.782	0.637
MDJNMF	0.725	0.778	0.630
SPID-MDJNMF	0.715	0.752	0.659
JNMF	0.671	0.782	0.659
SNF-JNMF	0.702	0.782	0.656
OC-JNMF	0.679	0.812	0.648
JSNMF	**0.851**	**0.812**	**0.690**

## Discussion and conclusion

The JSNMF algorithm integrates multimodal tumor data such as cellular features, pathway scores, and gene expression data from pathology images to investigate biological processes linked with tumor recurrence. This method enables us to utilize prior information from various data sources and obtain biologically interpretable features. Compared to previous studies, this is the first multimodal data fusion study to incorporate pathway information of tumor. Our research measures the link between biological pathways consisting of multiple genes and cellular features in tumor. This is done to broaden the comprehension of the methods by which genetic molecules affect cellular features.

Compared with existing matrix factorization methods and deep learning methods, JSNMF not only exhibits excellent performance in matrix reconstruction but also achieves superior biological analysis results. Its advantages stem from the following aspects. First, targeting similarities in data patterns across different modalities, this study employs the SNF algorithm for feature fusion of the feature matrices from three data sources. Subsequently, it extracts prior knowledge from the fused matrix using the PCA algorithm and embeds it into the objective function of JSNMF. The introduction of prior knowledge significantly enhances the clinical applicability of the shared data matrix derived from the analysis of the basis matrix $W$. On the other hand, analysis of shared modules using the three coefficient matrices $H$ reveals that the prior information extracted by the SNF algorithm can significantly strengthen the correlations among various features within the shared modules. Therefore, from an algorithmic perspective, the JSNMF algorithm with multiregularization term constraints enables superior model factorization performance. From a biological perspective, SNF preserves the complete biological information of multimodal data prior to matrix factorization, avoiding the loss of biological information during data fusion. This, in turn, ensures that JSNMF outperforms other comparative methods in both tumor prognosis analysis and cross-modal data association tasks.

Extracting clinically relevant feature association patterns from the co-modules is a common approach in matrix factorization algorithms. Take the SARC dataset as an example, this study identified co-module 2 as a representative module related to recurrence by calculating the correlation between clinical indexes and the basis vectors of the co-modules. Subsequently, significantly distinct cellular features, pathways, and genes were obtained. The enrichment analysis indicated a significant difference in genes between the recurrence and control groups. These genes were mainly found to be enriched in pathways that overlapped with the featured pathways in the module under all three pathway background information, including PI3K/AKT signaling in cancer, ERBB4 pathway, which plays important roles in tumor development. Hyperactivation of PI3K/Akt signaling is a vital process for cell proliferation, survival, and metabolic homeostasis in human cancers [43]. *In vivo*, ERBB4 boosts tumor invasion and metastasis, while ERBB4 knockdown stops these effects, indicating its potential as a therapeutic target [44].

In addition, cellular characteristics associated with relapse primarily comprise the nucleus’s short axis, area, and the ratio of the long axis to the short axis. The relapse samples demonstrated smaller nuclei in terms of area, a significantly shorter short-axis, and a smaller long-to-short-axis ratio, implying that relapse cell shapes were more circular and less red tinted. Based on previous research [45], it is possible that the morphology of certain white blood cells like lymphocytes, monocytes, and granulocytes could align with this description, indicating a greater quantity of immune cells in recurrent tissues. Additionally, correlation analysis demonstrated that differentially expressed genes were notably and negatively correlated with significant cellular features. Furthermore, the gene set was primarily enriched for pathways related to tumors and less related to the immune system. Correlation analysis revealed a significant negative association between the FSR2 gene and all four cellular features, implying that FSR2 was upregulated in irregular cells. According to high-resolution genomic mapping, FSR2 was amplified in liposarcoma [46]. FSR2-closely related fibroblasts’ cytosol exhibited a pike-shaped or irregularly triangular cytosol, with a central ovoid nucleus, protruding cytoplasm, and radial shape when growing. This may indicate that the cells in the nonrelapsed samples are more irregular. Incorporating pathway information allows for interpreting genetic changes within the context of biological pathways and changes in cellular characteristics.

Moreover, the JSNMF model functions to mine potential biomarkers related to relapse by utilizing representative co-modules. In this study, we analyzed several genes using ROC analysis in the co-module to identify recurrence samples independently. The identified genes include PAPPA, MDM2, and CAMK1D, which previous studies have linked to sarcoma or immune response. PAPPA enhances the role of IGF-1 signaling, promoting immune escape [47]. Meanwhile, chromatin-bound MDM2 control of cellular metabolism is an essential factor in liposarcoma development. Abnormal enhancement of MDM2 activity, including MDM2 gene amplification and overexpression, inhibits the function of the p53 protein and reduces its oncogenic role. MDM2 gene amplification is also found to be more pro-oncogenic [48–50]. CAMK1D, expressed in tumors refractory to anti-PD-L1 treatment, quickly inhibits the terminal apoptotic cascade through T cell recognition, and may contribute to tumor immune tolerance [51]. Predictor variables constructed using these three genes had an AUC exceeding 0.8. This indicates that the JSNMF algorithm accessed molecular characteristics associated with sarcoma recurrence or immune response.

Despite the effective improvements in search efficiency and biological plausibility of JSNMF achieved by introducing prior knowledge, this study still has limitations. First, although the proposed method has been experimentally validated in studies on multiple common tumors, the current sample size remains limited, and it fails to fully cover all tumor subtypes. This, to a certain extent, restricts in-depth exploration of whether JSNMF can effectively capture the variability in recurrence patterns across different subtypes. Second, the prior knowledge of JSNMF is derived solely from the data itself; its data applicability requires careful consideration when applied to different clinical scenarios (e.g. datasets generated by different pathological image scanning platforms or different gene sequencing technologies). Since different detection technologies may induce differences in data distribution, which in turn affect the consistency of SNF-based feature fusion and the performance stability of JSNMF, further validation in multicenter and multidetection-platform datasets is required to enhance the clinical translational value of the results. To address the above limitations, future studies will explore the application potential of JSNMF in investigating subtype-specific tumor recurrence mechanisms by expanding the sample size and extending the algorithm to more tumor subtypes.

**Key Points**

- This study propose a data-driven joint similarity nonnegative matrix factorization (JSNMF) model. By incorporating pathway score data alongside tumor pathological images and gene expression data, this model enhances the interpretability of associations among multimodal data.
- The JSNMF model employs the similarity network fusion model to compute a fusion matrix for three-modal data labeled by tumor recurrence. Additionally, it calculates prior information and incorporates it into a JNMF model with network regularization constraints.
- By integrating prior knowledge-based network regularization constraints and orthogonal constraints, the model enhances matrix factorization performance and associative capabilities.
- The bioinformatic analysis indicated that the model could identify potential biomarkers related to the infiltration level of immune cells for diagnosis of recurrence.

## Supplementary Material

Supplementary_materials_bbaf577

## Data Availability

JSNMF code can be found at https://github.com/Jindsmu/JSNMF.
