# Supplementary material for: Joint similarity nonnegative matrix factorization model for identification of recurrence-related association patterns in tumor
Source: Brief Bioinform. 2025 Nov 3;26(6):bbaf577. doi: 10.1093/bib/bbaf577 (PMC12581850; doi:10.1093/bib/bbaf577)
Supplement: Supplementary_materials_bbaf577 [file supplementary_materials_bbaf577.pdf]

Supplementary materials

Joint similarity non-negative matrix factorization model for identification of recurrence-related association patterns in tumor

Jin Deng[1], Junjie Lan[1], Ruolan Du[1], Tao Xu[1], Lechun Liu[1], Kaihan Huang[1], Lin Chen[2], Yongwei Zhang[1]*

[1] *College of Mathematics and Informatics, South China Agricultural University, Guangzhou 510642, China.*

[2] *Department of General Practice, Sun Yat-Sen Memorial Hospital, Guangzhou 510120, China.*

* *Corresponding Author*

1. The computational complexity of the JSNMF algorithm

The complexity of computational algorithms is an important method for evaluating algorithm performance. The complexity of algorithms is mainly divided into time complexity and space complexity. The objective function is as follows:

$$f\left(W,H_I\right)=\min\left[\sum_{I=1}^{3}\left(\left\|X_I-WH_I\right\|_F^2+\beta\left\|H_IH_I^T-I\right\|_F^2\right)+\alpha\left\|W-R\right\|_F^2\right] \tag{1}$$

The computational cost of JSNMF algorithm mainly focuses on the following two update steps:

$$W_{ij}\leftarrow W_{ij}\frac{\left(\sum_{I=1}^{3}X_IH_I^T+\alpha R\right)_{ij}}{\left(\sum_{I=1}^{3}WH_IH_I^T+\alpha W\right)_{ij}} \tag{2}$$

$$H_{Iij}\leftarrow H_{Iij}\frac{\left(W^TX_I\right)_{ij}}{\left(W^TWH_I+\beta\left(H_IH_I^T-E\right)H_I\right)_{ij}} \tag{3}$$

We assume that $X_I\in R^{n\times q_I}$ and rank=k, thus obtaining $H_I\in R^{q_I\times k}$, $W\in R^{k\times n}$ and $R\in R^{k\times n}$.

Firstly, calculate the time complexity.

The time complexity of the update functions for *W* and *H* are as follows:

$$O\left[(nk\sum_{I=1}^{3}q_I)+(nk\sum_{I=1}^{3}q_I)+(kn)\right]\approx O(nk\sum_{I=1}^{3}q_I) \quad (4)$$

$$O\left[(nk\sum_{I=1}^{3}q_I)+(k^2\sum_{I=1}^{3}q_I+k^2\sum_{I=1}^{3}q_I)+(kn\sum_{I=1}^{3}q_I)\right]\approx O(nk\sum_{I=1}^{3}q_I+k^2\sum_{I=1}^{3}q_I) \quad (5)$$

The highest order term that combines all update steps:

$$T_{\text{iter}}=O(nk\sum_{I=1}^{3}q_I)+O(nk\sum_{I=1}^{3}q_I)+O(k^2\sum_{I=1}^{3}q_I)=O(nk\sum_{I=1}^{3}q_I+k^2\sum_{I=1}^{3}q_I) \quad (6)$$

Assuming the JSNMF runs T iterations, the time complexity of JSNMF as follows:

$$T_{\text{total}}=t\times O(nk\sum_{I=1}^{3}q_I+k^2\sum_{I=1}^{3}q_I)=O(tnk\sum_{I=1}^{3}q_I+tk^2\sum_{I=1}^{3}q_I) \quad (7)$$

Secondly, calculate the spatial complexity.

Based on the dimensions of each data structure, we can obtain the storage space occupied by each data structure, as shown in Table 1.

During the iterative update process, the temporary matrix needs to be stored separately, as shown in Table 2.

Table 1: Main data structures for storage.

Data structure	X_I	H_I	W	R	E
Dimension	$n\times q_I$	$k\times q_I$	$n\times k$	$n\times k$	k^2
Storage space	$n\sum_{I=1}^{3}q_I$	$k\sum_{I=1}^{3}q_I$	$O(nk)$	$O(nk)$	$O(k^2)$

Table 2: Peak memory requirement for intermediate variables.

Iterative update	W	H_I
intermediate variable	WH_I	W^TX_I, $H_IH_I^T$, W^TW
Space complexity	$O(n\sum_{I=1}^{3}q_I)$	$O(k\sum_{I=1}^{3}q_I)$, $O(k^2)$, $O(k^2)$

The peak memory of resident data and intermediate variables needs to be stacked for calculation:

$$S_{total}=O(2n\sum_{I=1}^{3}q_I+2k\sum_{I=1}^{3}q_I+2nl+3k^2) \quad (8)$$

2. Supplementary table

Table S1: Comparison of effects of deep learning methods in three datasets.

	Methods	JSNMF	SGEGCAE	MOGLAM	OmiEmbed
SARC	Time	**1.438**	13.903	121.994	2756.675
	Relative error	**0.884**	1.134	3.001	1.608
	Euclidean distances	66.504	40.061	189.128	**34.213**
	Corr (X1, XX1)	**0.907**	0.620	-0.070	0.589
	Corr (X2, XX2)	**0.748**	0.711	0.004	0.347
	Corr (X3, XX3)	**0.680**	0.571	-0.002	0.074
TNBC	Time	**0.572**	20.258	113.430	3130.389
	Relative error	**0.559**	0.969	3.005	5.488
	Euclidean distances	**6.016**	10.998	66.001	32.997
	Corr (X1, XX1)	**0.995**	0.875	0.015	0.594
	Corr (X2, XX2)	**0.981**	0.894	-0.017	0.327
	Corr (X3, XX3)	**0.720**	0.540	-0.026	-0.007
KIRC	Time	**1.878**	16.590	129.228	3858.635
	Relative error	**0.669**	0.806	3.002	5.117
	Euclidean distances	38.254	**28.428**	240.001	90.239
	Corr (X1, XX1)	**0.993**	0.955	0.021	0.811
	Corr (X2, XX2)	**0.943**	0.890	-0.013	0.520
	Corr (X3, XX3)	**0.811**	0.708	-0.017	0.327

Table S2: Time complexity and space complexity of 8 algorithms.

Methods	Time Complexity	Spatial Complexity
SVD-JNMF	$O((2n+k)k\sum_{I=1}^{3}q_I+nk^2+2kq_1q_2$ $+2kq_1q_3+k(q_1^2+q_2^2+q_3^2))$	$O(2(n+k)\sum_{I=1}^{3}q_I+\sum_{I=1}^{3}q_I^2$ $+q_1q_3+q_1q_2+nk+3k^2)$
DSRJNMF	$O((2n+k)k\sum_{I=1}^{3}q_I+nk^2+2kq_1q_2$ $+2kq_1q_3+k(q_1^2+q_2^2+q_3^2))$	$O(2(n+k)\sum_{I=1}^{3}q_I+\sum_{I=1}^{3}q_I^2$ $+q_1q_3+q_1q_2+nk)$
JCB-SNMF	$O((2n+k)k\sum_{I=1}^{3}q_I+nk^2+2kq_1q_2$ $+2kq_1q_3+2kq_2q_3+k(q_1^2+q_2^2+q_3^2))$	$O(2(n+k)\sum_{I=1}^{3}q_I+\sum_{I=1}^{3}q_I^2$ $+q_1q_3+q_2q_3+q_1q_2+nk)$
OSJNMF-C	$O((n+kn+2k)k\sum_{I=1}^{3}q_I+nk^2+nk$ $+k(q_1q_2+q_1q_3+q_2q_3+q_1^2+q_2^2+q_3^2))$	$O(2(n+k)\sum_{I=1}^{3}q_I+q_1q_3$ $+q_2q_3+q_1q_2+4k^2+nk)$
scMNMF	$O((2n+k)k\sum_{I=1}^{3}q_I$ $+n(k^2+2dk)+2d^2(n+k))$	$O((n+2k)\sum_{I=1}^{3}q_I+2nk+$ $2nd+2dr+4k^2+2d^2)$
MDJNMF	$O((2n+1+k)k\sum_{I=1}^{3}q_I+nk^2)$	$O((2n+k)\sum_{I=1}^{3}q_I+$ $2q_1q_3+2nk+4k^2)$
SPID-MDJNMF	$O(2nk\sum_{I=1}^{3}q_I+2nk^2$ $+2k(q_1q_2+q_1q_3)+k^2\sum_{I=1}^{3}q_I)$	$O(2(n+k)\sum_{I=1}^{3}q_I+4k^2$ $+2nk+q_1(q_2+2q_3))$
JSNMF	$O(nk\sum_{I=1}^{3}q_I+k^2\sum_{I=1}^{3}q_I)$	$O(2(n+k)\sum_{I=1}^{3}q_I+2nk+2k^2)$

3. Supplementary figure

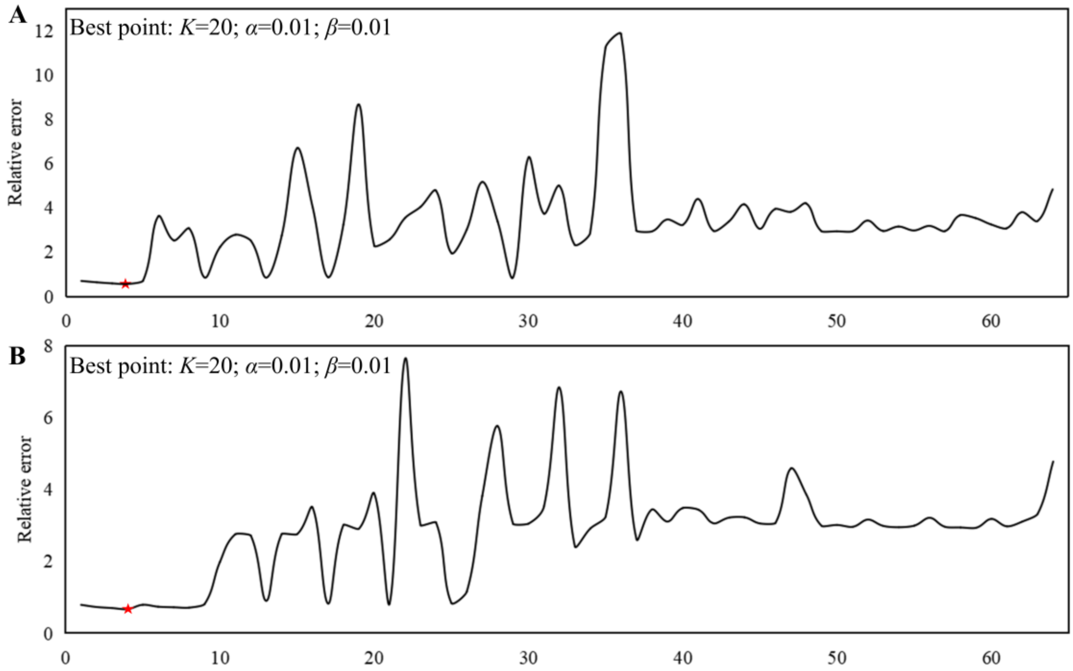

Figure. S1. The relative error of 64 combinations of three parameters: (A) TNBC dataset, (B) KIRC dataset.

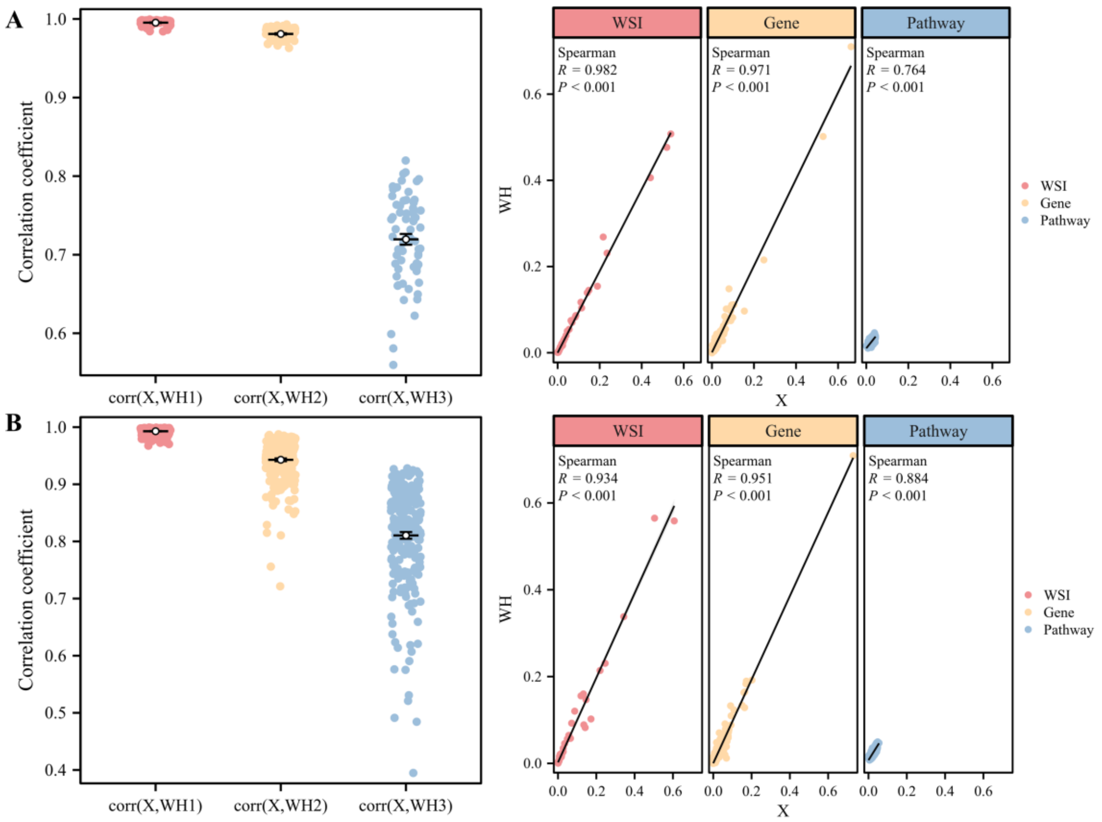

Figure. S2. The factorization effect of four datasets. The left graph shows Pearson correlation between *X* and *WH* in three types and the right graph shows the Pearson correlation between *X* and *WH* for the WSI, gene, and pathway. (A) TNBC dataset, (B) KIRC dataset.

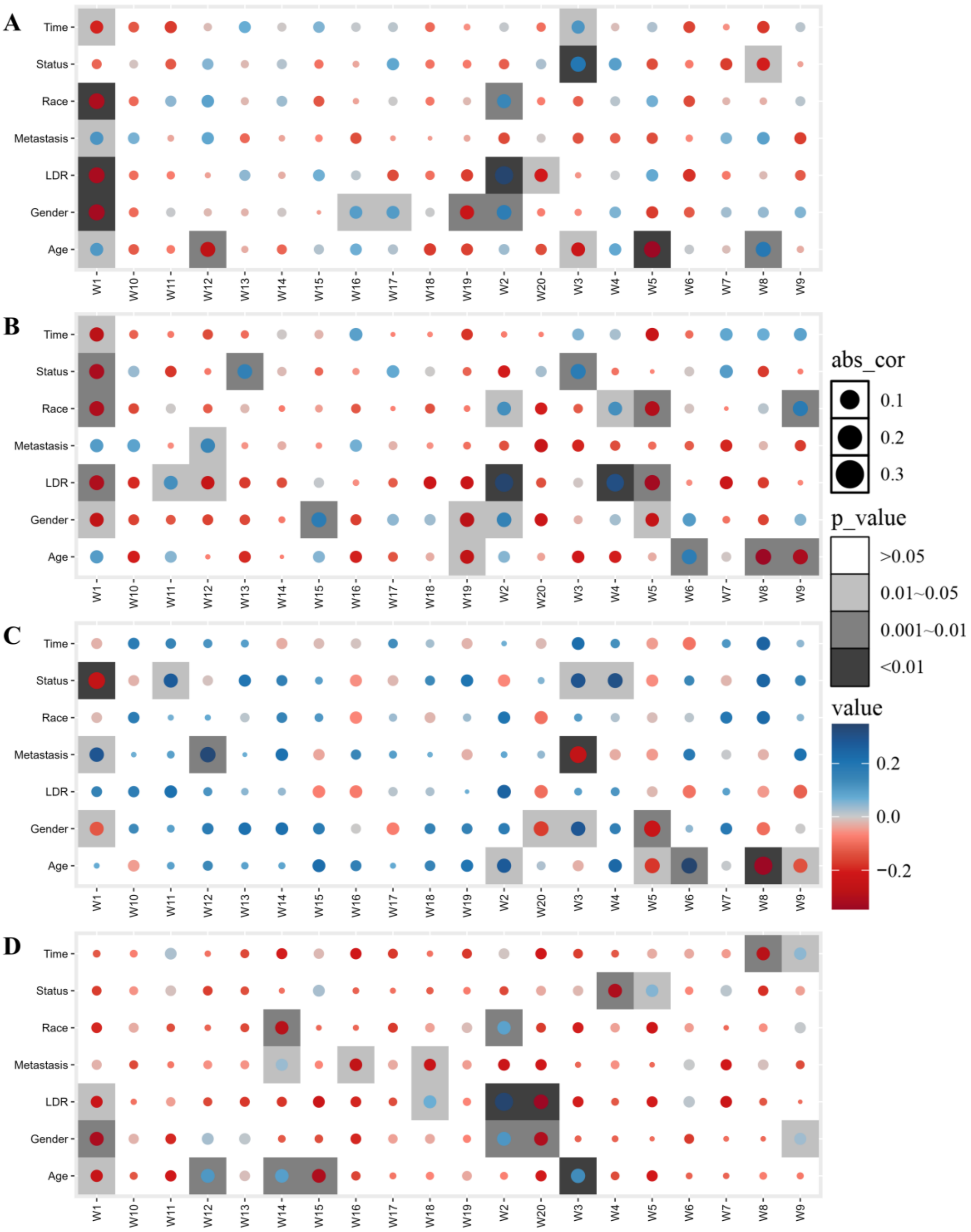

Figure. S3. Analysis of correlations between W matrices (calculated by JSNMF from different modality combinations) and seven clinical indicators based on the Pearson correlation coefficient: (A) WSI-gene-pathway; (B) WSI-gene; (C) WSI-pathway; (D) gene-pathway.

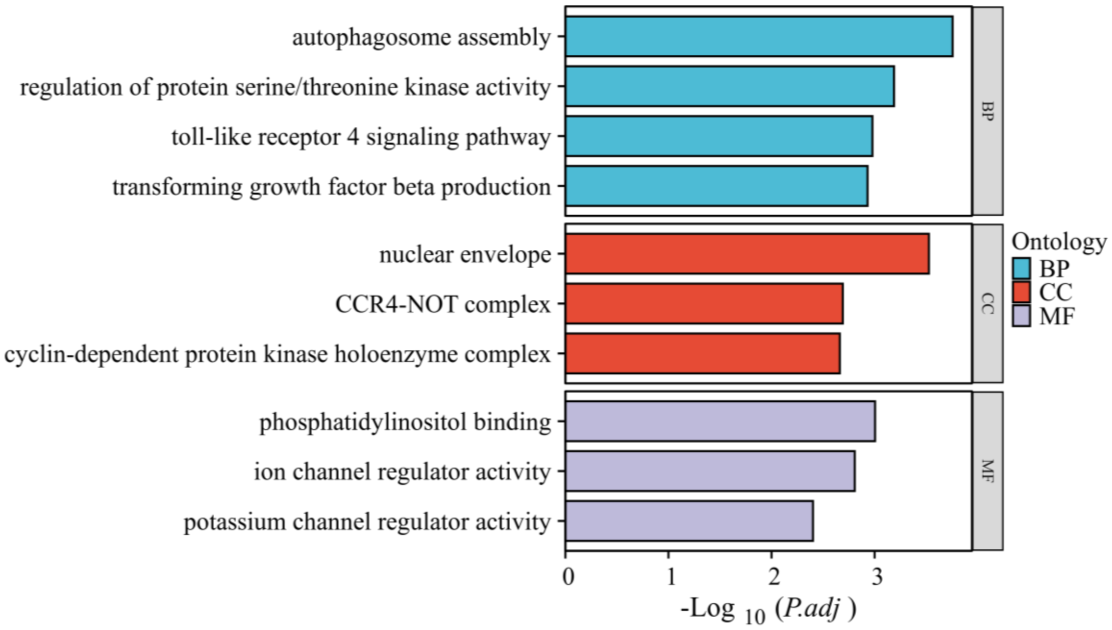

Figure. S4. The gene ontology analysis of genes mined by JSNMF in co-module 2.

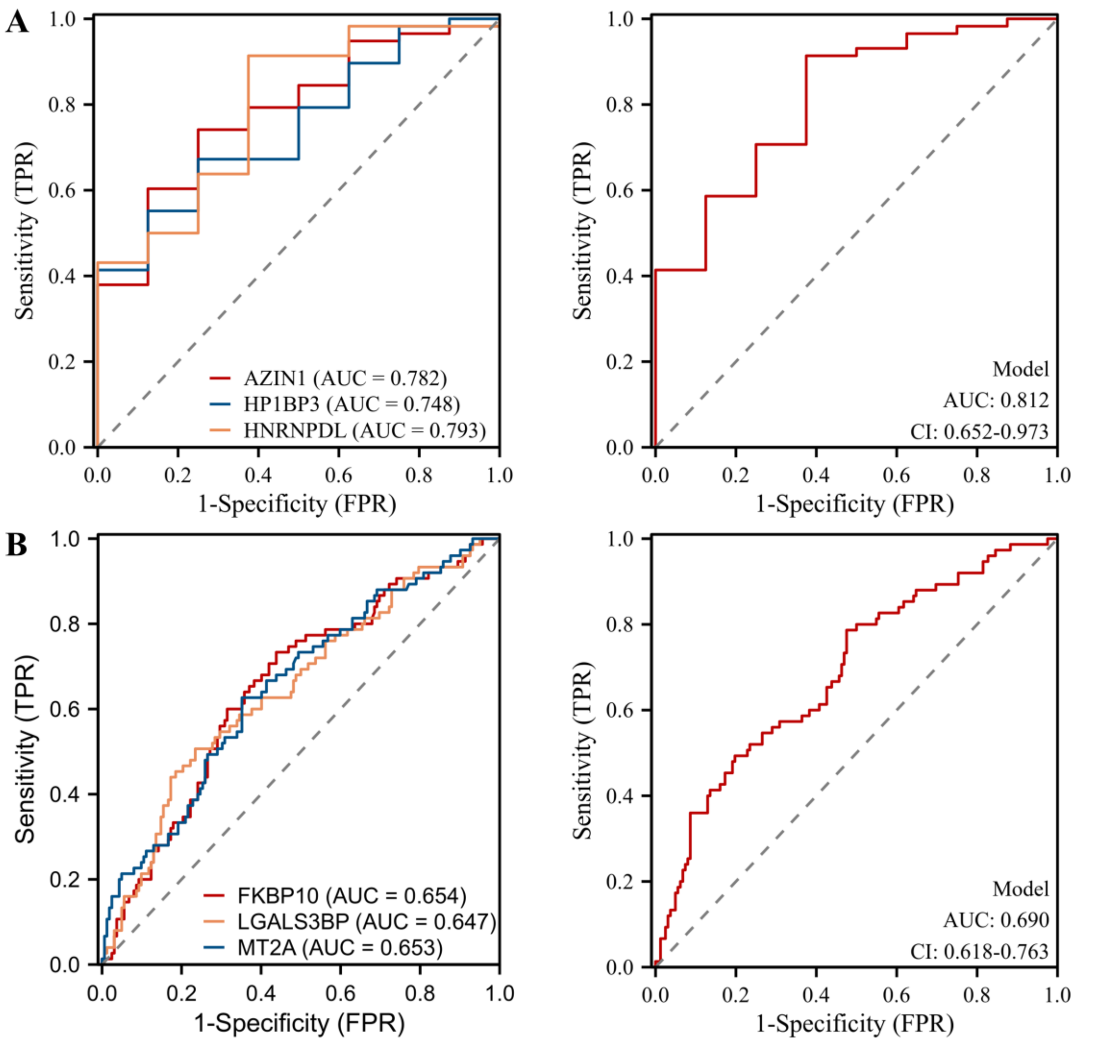

Figure. S5. Top 3 genes by AUC value screened by JSNMF in the (A) TNBC and (B) KIRC datasets, respectively (left figure), and predictive probability ROC curves of the top 3 genes based on the corresponding datasets (right figure).

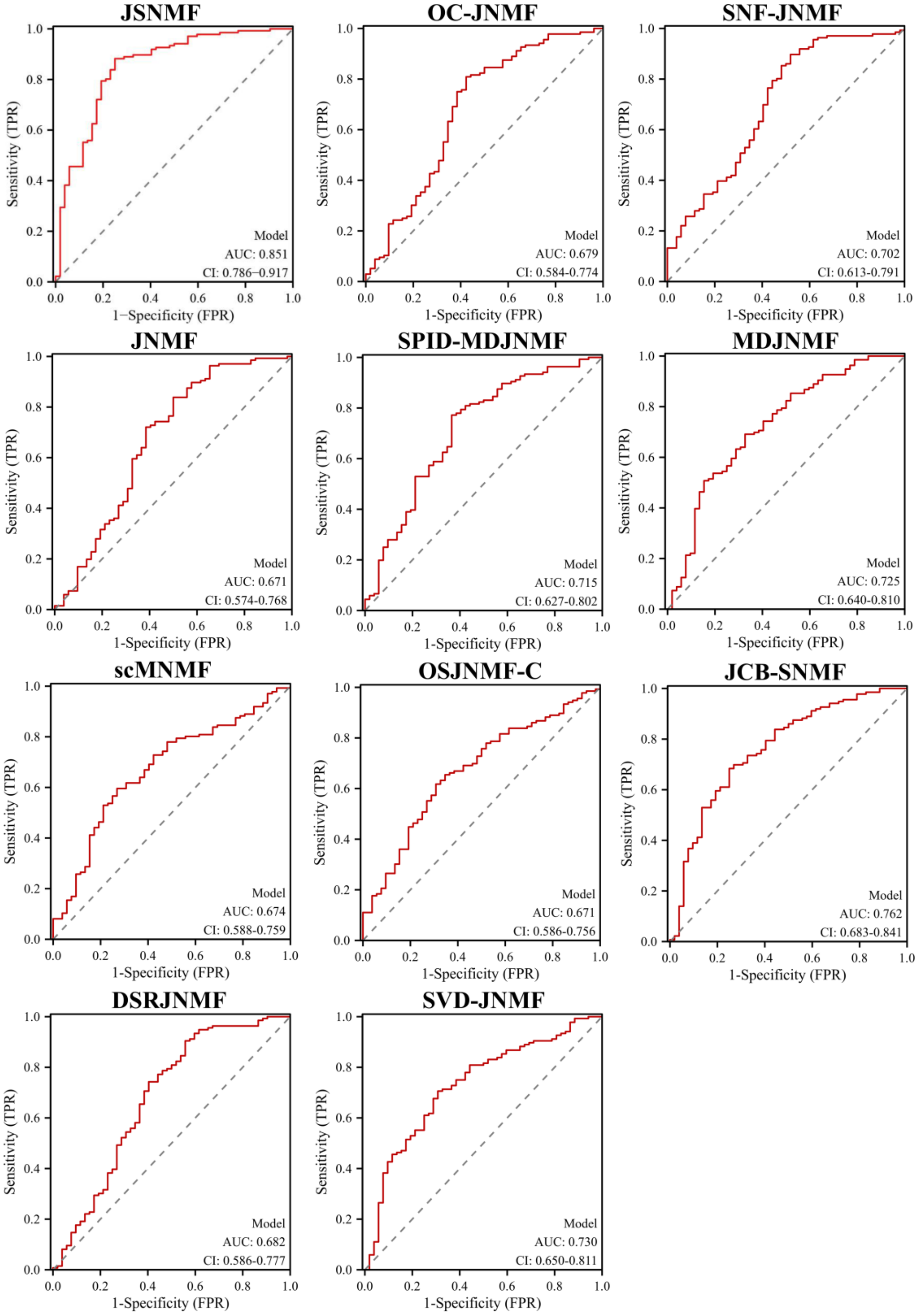

Figure. S6. Predictive ROC curves of 11 methods on the SARC Dataset.

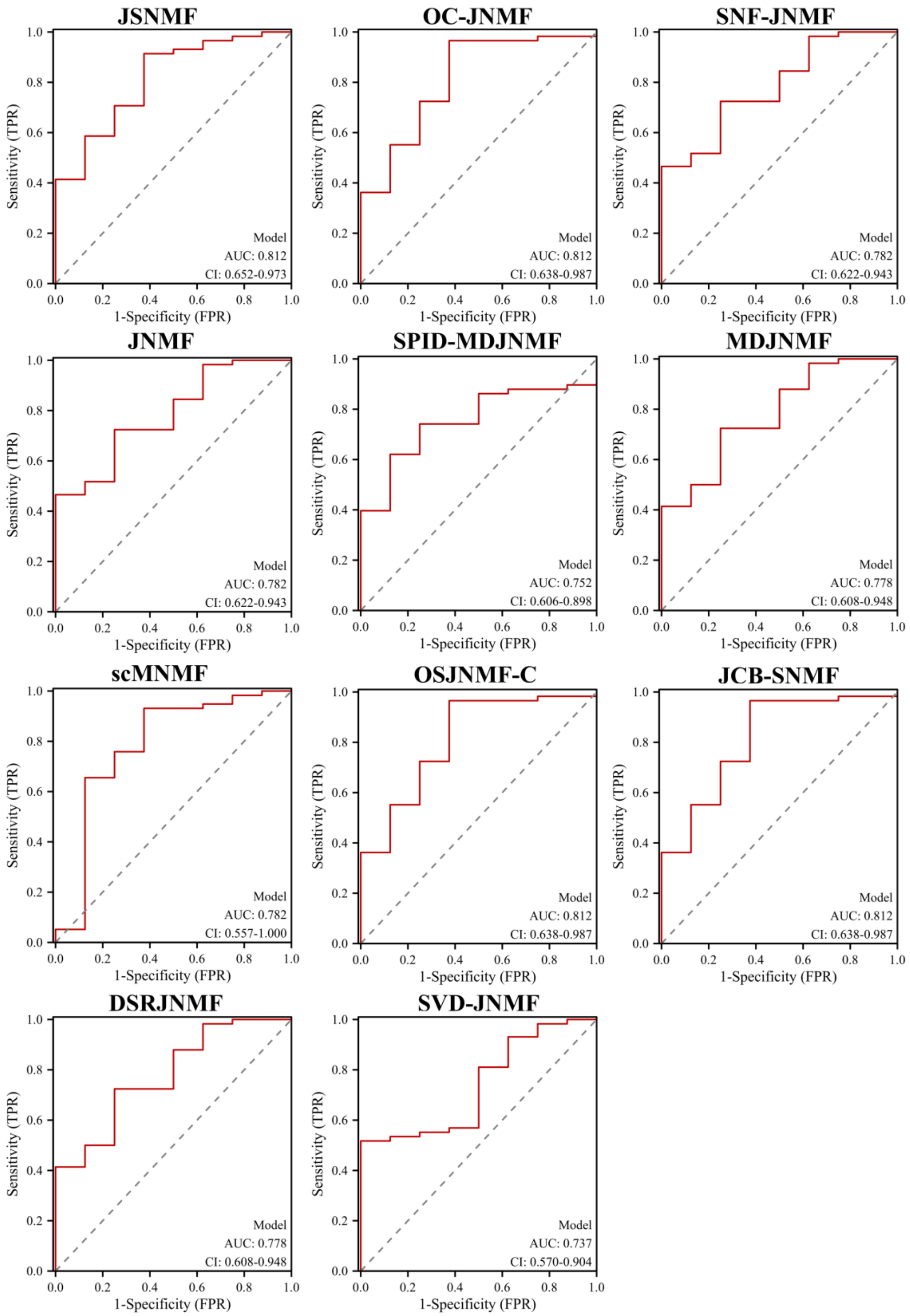

Figure. S7. Predictive ROC curves of 11 methods on the TNBC Dataset

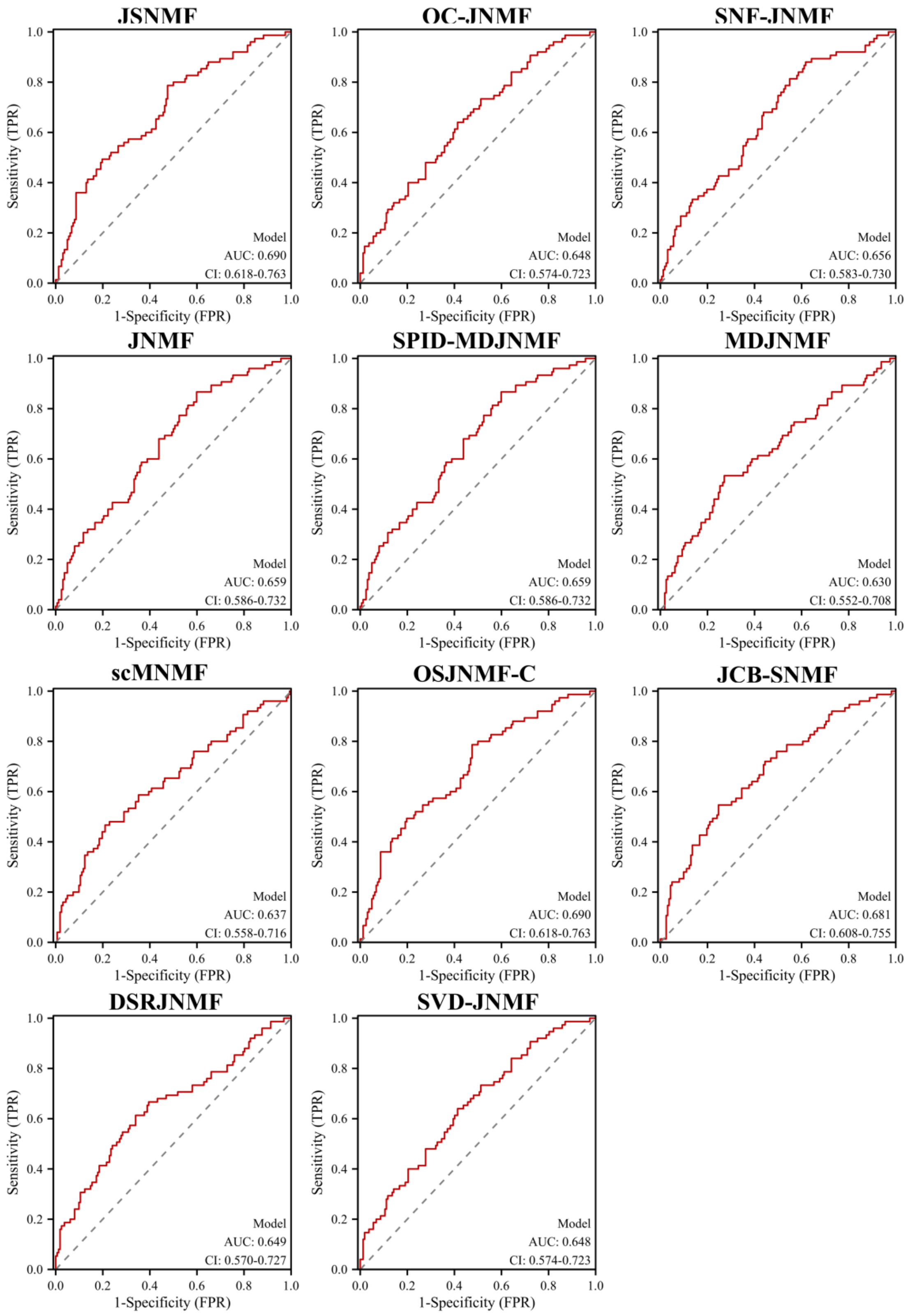

Figure. S8. Predictive ROC curves of 11 methods on the KIRC Dataset
